# Characteristics and causes in patients presenting with convulsions in the Danish prehospital setting

**DOI:** 10.1186/s13049-025-01532-x

**Published:** 2025-12-26

**Authors:** August Emil Licht, Johannes Bladt Andersen, Martine Siw Nielsen, Stig Alexander Larsen, Søren Mikkelsen

**Affiliations:** 1 The Prehospital Research Unit, Region of Southern Denmark, Odense University Hospital, Odense, Denmark; 2 Department of Regional Health Research, University of Southern Denmark, Odense, Denmark; 3 Department of Anesthesiology and Intensive Care Medicine, Kolding Hospital, Kolding, Denmark; 4 Department of Geriatrics, Slagelse Hospital, Slagelse, Denmark

## Abstract

**Background:**

Convulsing patients are one of the most common reasons for dispatch in the prehospital system. Determining the underlying cause of symptoms is paramount for both patient safety and determining the correct patient course. This study aims to describe the population of patients presenting with convulsions at first contact with an anesthesiologist-staffed mobile emergency care unit, to uncover the underlying reason for the convulsions, and to establish the outcome of the patient and correlate this to patient age.

**Methods:**

This retrospective cohort study was conducted through a manual review of prehospital records paired with data from the Danish Civil Person Register. It investigates all patients presenting with convulsions in Odense, Denmark, from January 1, 2011, through December 31, 2020.

Patients were stratified into seven age groups and had their diagnoses categorized into eight diagnostic groups. (Endocrine, Febrile convulsions, Cerebrovascular, Cardiopulmonary, Miscellaneous, Neurological, Psychiatric, and Non-specific). Patient mortality was recorded at 7, 30, and 90 days.

**Results:**

In total 3,388 patients were analyzed. The distribution of patients across age groups only stood out for patients aged 0–4 years, who accounted for 33% (*n* = 1,130) of the population. The underlying cause for convulsions varied greatly depending on patient age. The most common diagnoses were Unspecific diagnoses (38%, *n* = 1,289), Febrile convulsions (25%, *n* = 837), and Neurological diseases (20%, *n* = 689). The highest rate of conveyance with MECU physician escort was in patients within the age group (80 + years) with a conveyance rate of 44.1%. The lowest rate of anesthesiologist-escorted conveyance was found in patients within the age group (20–39 years) with a conveyance rate of 22.3%. Ninety-three patients died within 7 days of contact with the mobile emergency care unit. At thirty days, 147 (4.3%) had died, and the total number of deaths within 90 days was 203 (6.0%).

**Conclusion:**

Patient trajectories vary greatly across age groups. Patient mortality rates following an episode of convulsions increased considerably with age. Insight in underlying causes of convulsions and their severity may help the prehospital physician determine the correct course of action for the patient, benefiting both the provider and the patient.

## Introduction

Prehospital convulsions are among the most common reasons for dispatching ambulances [1, 2]. Convulsions have a wide range of possible underlying causes ranging from benign self-resolving epileptic episodes to cerebral hypoxia due to cardiac arrest [3–5]. This wide range of possible scenarios necessitates knowledge on the underlying causes of convulsions, as it is crucial in guiding the patient's treatment and trajectory [6–8].

Current gaps in knowledge may lead to preconceived notions in prehospital personnel [4]. Even professional care providers may be delaying treatment when handling patients with dramatic episodes of convulsions. Although status epilepticus is an emergency, prompt treatment of patients with status epilepticus is challenging [9]. Moreover, other possible underlying causes include stroke, structural brain damage, traumatic injuries, anoxic brain injury, and neuroinfections [10]. Provoked convulsions may be triggered by factors such as withdrawal symptoms, electrolyte imbalances, hypoglycemia [11], or extreme stress such as sleep deprivation and hyperventilation [12, 13]. Non-somatic causes may also cause convulsions, including psychogenic non-epileptic convulsions [14, 15]. Currently, the highest level of dispatch in Denmark is consistently warranted for patients suspected of convulsing due to the risk of symptoms developing into life-threatening medical emergencies where the prognosis is highly dependent on the urgency of the treatment [16]. Sudden unexpected death in epilepsy is still one of the leading drivers of premature mortality in people with epilepsy [17].

Thus, mental preparedness is a vital component even for professional care providers. Studies indicate that EMS providers who mentally rehearse potential tasks before arrival report less on-scene stress and greater confidence, subsequently improving decision-making [18, 19] and handling of bystanders and first responders who may be distressed due to the violent presentation of convulsions [5].

Current gaps in knowledge of which patients present with certain causes of convulsions inhibit personnel in identifying the correct patient pathway and ensuring optimal safety and efficient use of healthcare resources [4, 20]. The aim of this study was to describe the population of patients presenting with convulsions at first contact with the prehospital anesthesiologist-staffed mobile emergency care unit, to identify the underlying cause of the convulsions, and to determine the patient's outcome and its correlation with age.

## Methods

This population-based single-center retrospective cohort study was conducted in accordance with the STROBE guidelines for observational studies [21]. It included patients treated by the prehospital anesthesiologist-staffed mobile emergency care unit (MECU) in Odense, Denmark, in the period from 01/01–2011 to 31/12–2020.

### System setting

This study was conducted at the MECU in Odense in the Region of Southern Denmark. The MECU in Odense covers an area of approximately 2.500 km^2^, servicing a population of approximately 300.000 people.

The average number of MECU missions is 10.5 per day, and the dispatch of the MECU is initiated either by the emergency medical dispatch center or following a secondary request by ambulance personnel having contact with a patient [22].

The MECU is part of the third and final tier of the Danish emergency medical service (EMS). In Odense, the MECU is always manned by a board-certified physician specialized in anesthesiology, assisted by a Paramedic.

The Danish EMS is tax-funded, ensuring that patients incur no immediate expenses for prehospital and in-hospital treatment [16, 23].

All patients receiving medical treatment in Denmark are assigned a unique 10-digit Civil Person Register (CPR) number, which enables correct identification and linkage to existing medical records [24, 25].

#### Inclusion criteria

- Patients in whom the dispatch criterion included convulsions
- Patients treated by the MECU where the prehospital diagnosis included convulsions
- Patients treated by the MECU whose electronic medical records or prehospital case presentations included the word convulsions

#### Exclusion criteria

- Patients residing outside of the region of Southern Denmark at the time of the incident
- Patients having an invalid or temporary civil person register number
- Incorrect patient registration in the MECU database
- MECU contacts in which the prehospital unit was cancelled before arrival at the scene

### Data sources, patient variables and outcomes

Data sources included prehospital medical records from the MECU, in-hospital medical records, the Danish National Patient Register [25] accessed through Statistics Denmark [26].

Patient age and sex were sampled. Additionally patient outcome variables: age, sex, conclusion of MECU contact, prehospital diagnosis, in-hospital diagnosis, and time of death were sampled.

### Data handling

A dataset was created based on the MECU medical records. All medical charts containing the word “convulsion” entered as either the dispatch criteria [27] and/or within the free field text notes of the prehospital medical record system (as entered by either the emergency medical technician, the paramedics, or the prehospital anesthesiologist) were collected [23]. Furthermore, all prehospital medical records with patients assigned a prehospital diagnosis associated with convulsions were collected (WHO ICD-10 Classification system diagnosis codes: R56.0, R56.8, G40.0—G40.9, G41.0—G41.9) [28].

All medical records were subsequently manually scrutinized by authors AEL and JBA to ensure that inclusion and exclusion criteria were met. Many patient contacts were represented by both dispatch criteria and free text fields, as well as prehospitally assigned diagnoses. Therefore, finally, the dataset was merged into records representing individual patient trajectories.

Using the patients’ CPR numbers and their time of contact with the MECU, the medical records were matched with the data in the Danish CPR register. This allowed for matching the prehospital data with the in-hospital diagnoses assigned to the patients following a full diagnostic work-up.

In cases where patients were admitted to the hospital for more than ten days, the latest assigned diagnosis was used. The most accurate in-hospital diagnosis after diagnostic workup was defined as the diagnosis recorded in the hospital discharge letter. In cases where the patient was not transported to the hospital, the prehospital diagnosis assigned to the patient by the anesthesiologist on duty at the MECU was used for analysis. The Danish legal regulations related to the General Data Protection Regulation prohibit the display or publication of observations from four or fewer individuals. Therefore, in all cases where the number of observations was below five patients, the numbers were anonymized using the term “ < 5″ “less than 0.4%” [29].

### Diagnostic groups

Patient were divided into 7 diagnostic groups according to the WHO ICD-10 Classification system diagnosis chapters. Febrile convulsions was assigned its own group. Chapter IX was split into two groups to discern cerebrovascular diseases from cardiovascular diseases. All diagnoses not contained within the first 6 groups were assigned to the miscellaneous group:

- Non-specific diagnoses (R00-R99 excluding R56.0)(Chapter XVIII)
- Febrile convulsions (R56.0 Exclusively)
- Neurological (G00-G99)(Chapter VI)
- Psychiatric (F00-F99)(Chapter V)
- Endocrine (E00-E90)(Chapter IV)
- Cerebrovascular (I60-I99)(Chapter IX)
- Cardiopulmonary (I00-I59)(Chapter IX)
- Miscellaneous (All not in other chapters)

### Statistical analysis

Non-parametric statistics were applied, as data were not expected to follow a normal distribution. Data are presented as proportions with 95% confidence intervals (CI) based on binomial distribution, or medians and quartiles. Data were analyzed with the Kruskal–Wallis test to describe uneven distribution among diagnostic groups.

Data were handled using Microsoft Excel, Redmond, Washington, USA, and Stata 17.0, StataCorp, College Station, Texas, USA.

### Ethics approval

This study was approved by the Judicial Office of the Region of Southern Denmark (Ref. No. 22/27227). According to the Act on Processing of Personal Data, in register-based studies approved by the regional Judicial Offices, no consent is required to use data already entered in the registry. Thus, no further approvals are necessary according to Danish law [30]. In addition to the required approvals, all data handling was carried out in accordance with Danish and European legislation concerning person-identifiable data [31, 32].

## Results

**Fig. 1:**
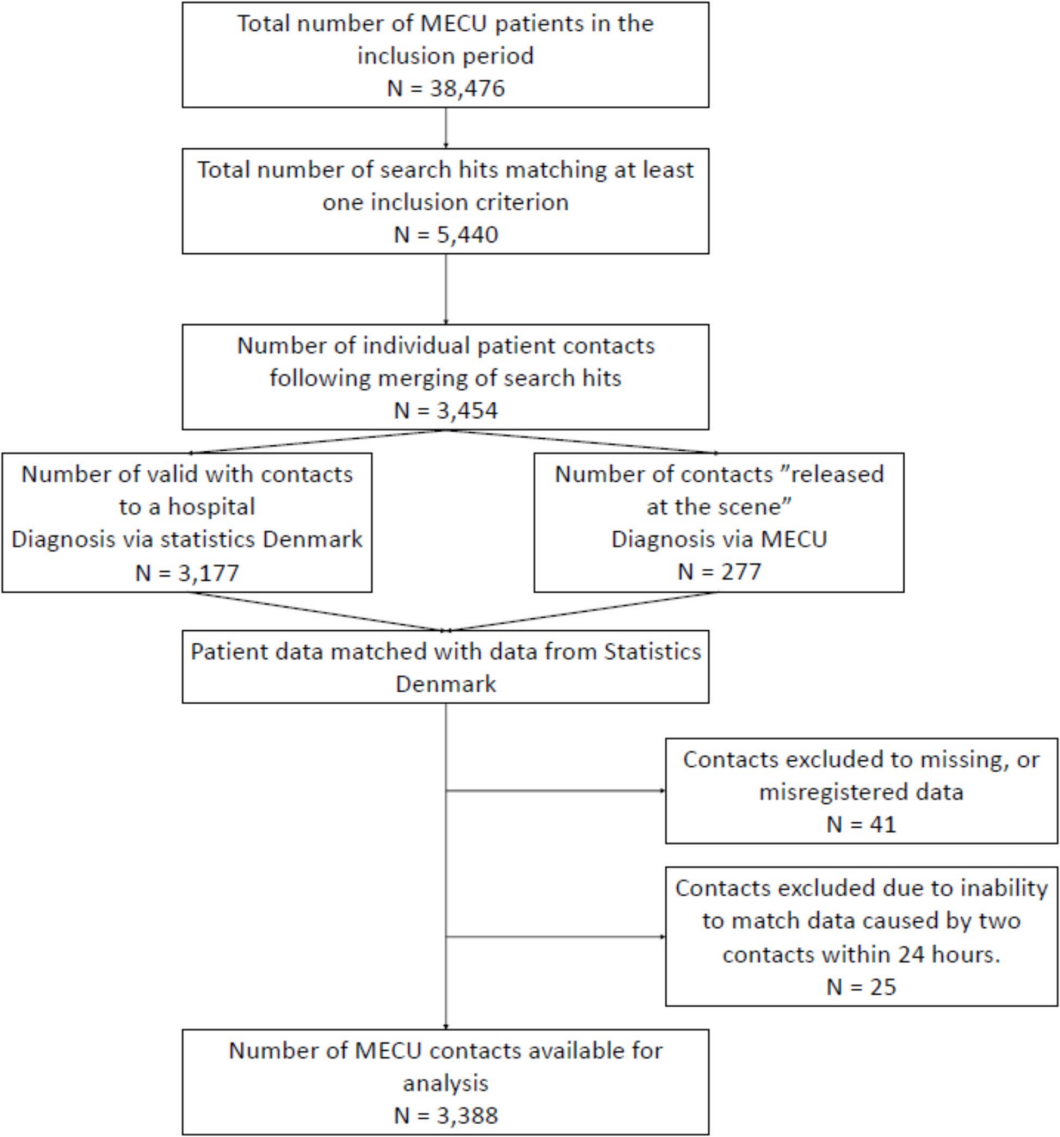
Study flowchart

During the observation period of ten years, the MECU treated 38,476 patients. A total of 5,440 entries of dispatch criteria, free text fields, or prehospitally assigned diagnoses were identified. Following the merging of medical records so that each record represented one single patient on one specific day of prehospital contact, a total of 3,454 patients were identified as having been treated by the MECU for a condition that included convulsions. Twenty-five patients were subsequently excluded because they had more than one registered MECU contact within 24 h of initial dispatch, which made it impossible to correctly match the prehospital data with the data from Statistics Denmark; thus, the analysis was not possible. None of the 25 excluded patients died within the observation period. In 41 patients, data were missing to such an extent that analysis was impossible. Thus, 3,388 patients were eligible for analysis. For the Flowchart, see Fig. 1.

**Fig. 2:**
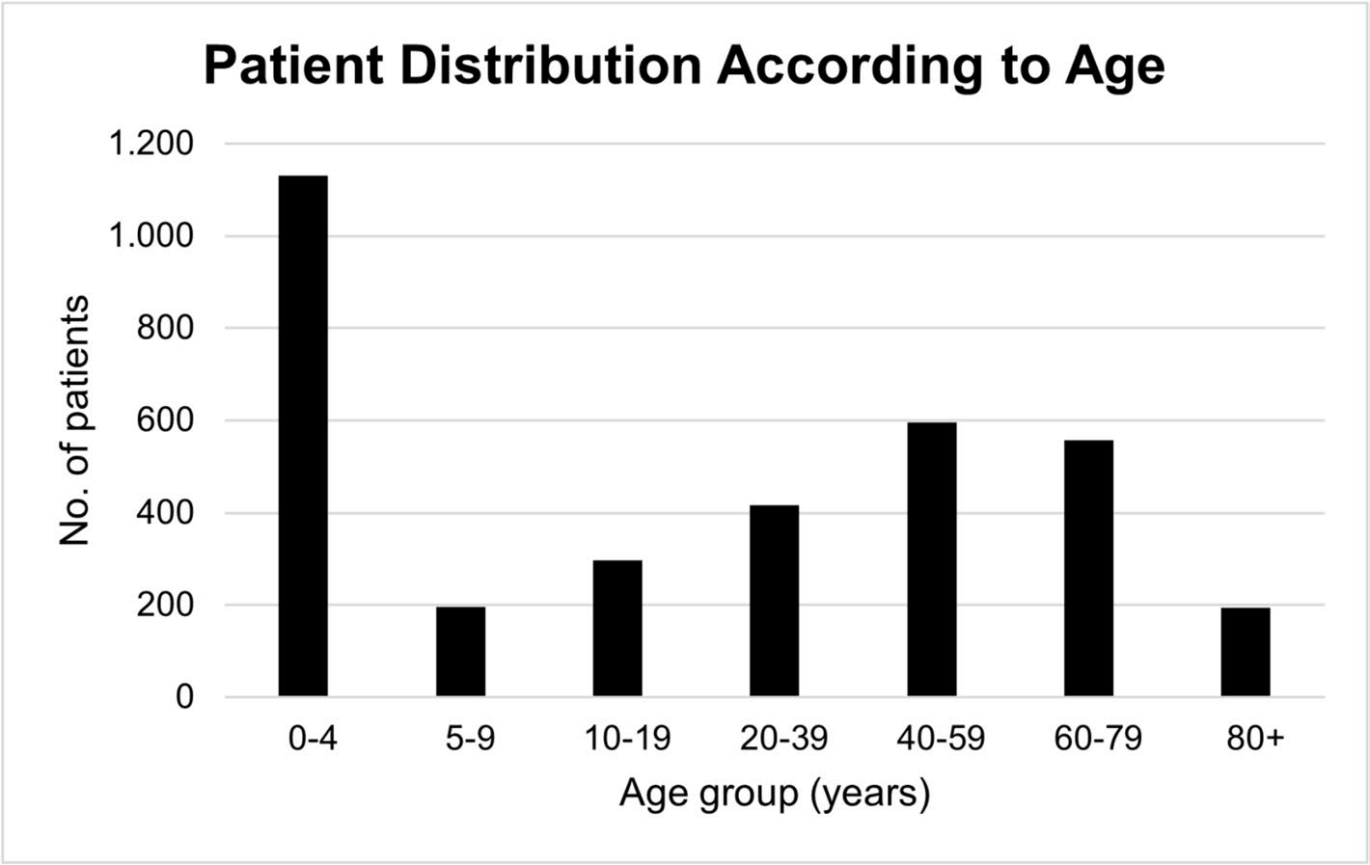
Age distribution among convulsing patients

The median age was 22 years (IQR 2—57). A total of 1,414 patients were female (42%), and 1,974 were male (58%). The age distribution of the included patients is depicted in Fig. 2*,* stratified into age intervals.

The 3,388 patients included for analysis were divided into seven common diagnostic groups and a “miscellaneous” group. The most frequent in-hospital diagnoses assigned to the patients were found within ICD-10 chapters XVIII (symptoms, signs, and abnormal clinical and laboratory findings, not elsewhere classified excluding Febrile convulsions (R560), (*n* = 1,289), Febrile convulsions (R560) (*n* = 837), and ICD-10 Chapter VI (Diseases of the nervous system) (*n* = 689) [28].

The largest group in the study was comprised of patients aged 0–4 years, representing 33.4% of the total population. Of these, 70% were diagnosed with febrile convulsions. The distribution of patients across the eight diagnostic groups is shown in Table 1. Kruskal–Wallis test demonstrated that groups differed significantly from each other *p* < 0.0001.

**Table 1:** Distribution of patients within eight selected diagnostic sub-groups

Diagnostic Groups (IDC-10 Chapter)	Non-specific (R00-R99)	Febrile Convulsions (R56.0)	Neurological (G00-G99)	Psychiatric (F00-F99)	Endocrine (E00-E90)	Cerebrovascular (I60-I99)	Cardiopulmonary (I00-I59)	Miscellaneous	Total
Age group (years)	N (%) [95%CI])	N (%) [95%CI])	N (%) [95%CI])	N (%) [95%CI])	N (%) [95%CI])	N (%) [95%CI])	N (%) [95%CI])	N (%) [95%CI])	
0–4	182 (16.1 [14.1—18.4])	794 (70.3 [67.5—72.9]	63 (5.6 [4.4—7.1])	0 (0.0 [0.0—0.3])	5 (0.4 [0.2—1.0])	0 (0.0 [0.0—0.3])	0 (0.0 [0.0—0.3])	86 (7.6 [6.2—9.3])	1130
5–9	69 (35.2 [28.9—42.1])	39 (19.9 [14.9—26.0])	68 (34.7 [28.4—41.6])	12 (6.1 [3.5—10.4])	0 (0.0 [0.0—1.9])	0 (0.0 [0.0—1.9])	0 (0.0 [0.0—1.9])	8 (4.1 [2.1—7.8])	196
10–19	180 (60.4 [54.8—65.8])	4 (1.3 [0.5—3.4])	79 (26.5 [21.8—31.8])	18 (6.0 [3.9—9.3])	11 (3.7 [2.1—6.5])	0 (0.0 [0.0—1.3])	0 (0.0 [0.0—1.3])	6 (2.0 [0.9—4.3])	298
20–39	218 (52.3 [47.5—57.0])	0 (0.0 [0.0—0.9])	126 (30.2 [26.0—34.8])	29 (7.0 [4.9—9.8])	11 (2.6 [1.5—4.7])	4 (1.0 [0.4—2.4])	8 (1.9 [1.0—3.7])	21 (5.0 [3.3—7.6])	417
40–59	299 (50.2 [46.2—54.2])	0 (0.0 [0.0—0.6])	139 (23.3 [20.1—26.9])	70 (11.7 [9.4—14.6])	31 (5.2 [3.7—7.3])	16 (2.7 [1.7—4.3])	10 (1.7 [0.9—3.1])	31 (5.2 [3.7—7.3])	596
60–79	258 (46.4 [42.3—50.6])	0 (0.0 [0.0—0.7])	162 (29.1 [25.5—33.0])	19 (3.4 [2.2—5.3])	22 (4.0 [2.6—5.9])	26 (4.7 [3.2—6.8])	17 (3.1 [1.9—4.8])	52 (9.4 [7.2—12.1])	556
80 +	83 (42.6 [35.8—49.6])	0 (0.0 [0.0—1.9])	52 (26.7 [21.0—33.3])	4 (2.1 [0.8—5.2])	5 (2.6 [1.1—5.9])	17 (8.7 [5.5—13.5])	12 (6.2 [3.6—10.4])	22 (11.3 [7.6—16.5])	195
Total	1289 (38.0 [36.4—39.7])	837 (24.7 [23.3—26.2])	689 (20.3 [19.0—21.7])	152 (4.5 [3.8—5.2])	85 (2.5 [2.0—3.1])	63 (1.9 [1.5—2.4])	47 (1.4 [1.0—1.8])	226 (6.7 [5.9—7.6])	3388

^*^Miscellaneous diagnoses are diagnoses within ICD10 Chapters: I, II, VII, VIII, X, XI, XII, XIII, XIV, XV, XVI, and XVI. Data are depicted as: N (% [95%CI]). % and 95% CI are defined by age group. Kruskal–Wallis: *p* < 0.0001

Two hundred seventy-seven patients (8.2%) were released at the scene by the MECU following treatment. Nine hundred forty-nine patients (28.0%) were transported to a hospital for further examination in an ambulance with escort by the MECU anesthesiologist. Two thousand eighty-nine patients (61.7%) were transported to the hospital by ambulance without MECU physician escort. Seventy-three patients (2.2%) had their MECU contact terminated in other ways, 65 patients because the MECU was reprioritized to patients elsewhere, and eight patients who were declared dead at the scene immediately upon the arrival of the MECU. The highest rate of conveyance with MECU physician escort was in patients within the age group (80 + years) with a conveyance rate of 44.1%. The lowest rate of anesthesiologist-escorted conveyance was found in patients within the age group (20–39 years) with a conveyance rate of 22.3%. The distribution of patient conveyance by age group is depicted in Table 2.

**Table 2:** Type of patient transport to the hospital

Transport type	Transported without MECU escort	Transported with MECU escort	No transport (released at the scene)	Other	Total
Age group (years)	N (%) [95%CI])	N (%) [95%CI])	N (%) [95%CI])	N (%) [95%CI])	
0–4	792 (70.1 [67.4—72.7])	254 (22.5 [20.1—25.0])	69 (6.1 [4.9—7.7])	15 (1.3 [0.8—2.2])	1130
5–9	109 (55.6 [48.6—62.4])	60 (30.6 [24.6—37.4])	22 (11.2 [7.5—16.4])	5 (2.6 [1.1—5.8])	196
10–19	177 (59.4 [53.7—64.8])	72 (24.2 [19.6—29.3])	42 (14.1 [10.6—18.5])	7 (2.3 [1.1—4.8])	298
20–39	269 (64.5 [59.8—68.9])	93 (22.3 [18.6—26.5])	46 (11.0 [8.4—14.4])	9 (2.2 [1.1—4.1])	417
40–59	350 (58.7 [54.7—62.6])	170 (28.5 [25.0—32.3])	59 (9.9 [7.8—12.6])	17 (2.9 [1.8—4.5])	596
60–79	303 (54.5 [50.3—58.6])	214 (38.5 [34.5—42.6])	25 (4.5 [3.1—6.6])	14 (2.5 [1.5—4.2])	556
80 +	89 (45.6 [38.8—52.6])	86 (44.1 [37.3—51.1])	14 (7.2 [4.3—11.7])	6 (3.1 [1.4—6.5])	195
Total	2089 (61.7 [60.0—63.3])	949 (28.0 [26.5—29.5])	277 (8.2 [7.3—9.1])	73 (2.2 [1.7—2.7])	3388

^*^Data are depicted as: N (%) [95%CI]). % and 95% CI are defined by age group. Kruskal–Wallis: *p* < 0.0001

Ninety-three patients died within 7 days of contact with the MECU. One hundred forty-seven died within 30 days, and the total number of deaths within 90 days was 203. The overall cumulative mortality rates were thus 2.7% at 7 days, 4.3% at 30 days, and 6.0% at 90 days. The lowest 90-day mortality rate was found in the age group (5–9 years), with no deaths within 90 days. The highest 90-day mortality rate was found in the age group (80 + years), with a mortality rate of 36%. The distribution of mortality across patient groups within the study is depicted in Table 3*.*

**Table 3:** Patient mortality

Mortality	7 days	30 days	90 days	Total No. of patients
Age group (years)	N (%) [95%CI])	N (%) [95%CI])	N (%) [95%CI])	
0–4	< 5 (< 0.4 [0.0—1.0])	< 5 (< 0.4 [0.0—1.0])	< 5 (< 0.4 [0—1.0])	1130
5–9	0 (0.0 [0.0—1.9])	0 (0.0 [0.0—1.9])	0 (0.0 [0.0—1.9])	196
10–19	< 5 (< 1.7 [0.0—3.9])	< 5 (1.7 [0.0—3.9])	5 (1.7 [0.7—3.9])	298
20–39	4 (1.0 [0.4—2.4])	6 (1.4 [0.7—3.1])	9 (2.2 [1.1—4.1])	417
40–59	19 (3.2 [2.1—4.9])	23 (3.9) [2.6—5.7]	34 (5.7 [4.1—7.9])	596
60–79	29 (5.2 [3.7—7.4])	60 (10.8 [8.5—13.6])	84 (15.1 [12.4—18.3])	556
80 +	36 (18.5 [13.6—24.5])	53 (27.2 [21.4—33.8])	71 (36.4 [30.0—43.4])	195
Total	93 (2.7 [2.2—3.4])	147 (4.3 [3.7—5.1])	203 (6.0 [5.2—6.8])	3388

^*^Data are depicted as: N (% [95%CI]). The percentage and 95% CI are calculated within each age group. Due to Danish GDPR laws, patient groups with less than five observations had to be grouped to keep the data anonymous. This is observed in the mortality of [0–4 years] and [10–19 years] age groups

Of the 3,388 patients included in the study, eight were declared dead at the scene immediately upon arrival of the prehospital physician without receiving resuscitation attempts. Twenty-eight other patients had cardiac arrest in the prehospital setting, of whom nineteen were successfully resuscitated. Nine patients developed cardiac arrest during conveyance to or upon arrival at the hospital. The initial prehospital diagnoses of these nine patients included conditions such as ventricular tachycardia, sepsis, and “drowning or near drowning”.

## Discussion

### Main findings

This study investigated 3,388 patients who presented with or developed convulsions in relation to contact with the MECU. Two out of five patients were assigned non-specific diagnoses during the subsequent in-hospital assessments. This emphasizes that convulsions are often merely a symptom rather than a distinct disease entity. Febrile convulsions accounted for one in four cases of patients with convulsions. Overall, neurological conditions constituted only one-fifth of the diagnoses assigned in this prehospital cohort of convulsing patients.

### Other studies

An English study of patients convulsing at contact with the EMS found that in 3.3% of all EMS contacts, convulsions had occurred [7]. A later study conducted on the same population identified epileptic convulsions as the most common cause for suspected convulsions in 74.7% of patients, but also concluded that patients with suspected convulsions generally represent a diagnostically heterogeneous group [8]. Although sudden unexpected death in epilepsy is one of the leading drivers of premature mortality in people with epilepsy [17], several studies have shown that life-threatening medical emergencies associated with suspected convulsions are uncommon [7, 8, 33].

A German study found that a large part of patients with suspected convulsions had high rates of transportation to the hospital without a documented medical reason (92%), which is identical to the conveyance rate found in this study. Thus, evidence-based guidelines are needed to help avoid unnecessary admissions [34]. Two studies have also addressed the need for the correct allocation of the EMS resources for optimal patient courses [34, 35]. In Finland, a non-conveyance protocol has been implemented, allowing EMTs to release well-recovered patients who have been suspected of having convulsions that have resolved at the scene [36]. Danish EMTs and paramedics are authorized to release patients at the scene after consulting with the MECU [37].

The ratio of male to female (58% to 42%) found in the present study correlates well with other studies [7, 33, 34]. While most similar studies do not report patient mortality data, the present study finds a mortality rate at 7 days of 2.7%. This is lower than reported by another study of prehospital convulsions seen at the hospital, which found a 7-day mortality of 4.6% [1]. Our study includes a large proportion of patients with febrile convulsions. This may account for the lower mortality in the study.

### Diagnostic groups

Non-specific diagnoses account for the most significant number of cases in this study. Non-specific diagnoses are increasingly common in the Danish EMS and hospital settings [38]. Although non-specific diagnoses may suggest less severe acute conditions, another Danish study in a similar setting reports a 6.0% 30-day mortality rate among patients discharged with non-specific diagnoses [39].

Only 20.3% of patients presenting with convulsions were assigned a diagnosis within the ICD-10 Chapter of Neurological Disease. However, in patients older than 4 years of age, neurological diagnoses account for 24% of all cases, with little variance across age groups. The vast majority of patients with neurological diseases were patients with epilepsy, whose condition was already known before EMS contact; epileptic patients, however, represent a diverse group of patients, with various syndromes and clinical symptoms [40]. In these patients, determining the correct course of treatment is especially helpful in avoiding unnecessary admissions to the hospital, thus preventing emergency room crowding [34, 41].

### Febrile convulsions

Febrile convulsions are the most common diagnosis in patients aged 0–4 years in the Odense MECU [42]. The present study found that febrile convulsions are the second most common diagnosis across all patients presenting with convulsions. The prehospital treatment of patients with febrile convulsions may differ from the treatment of other patients presenting with convulsions, as these cases are often uncomplicated convulsions that are often self-limiting. The patients may not require hospital admission. However, for parents, witnessing convulsions can be a distressing experience, potentially affecting their quality of life [43]. Therefore, counselling the parents often serves as an additional important function for the prehospital physicians [44].

### Octogenarians

This study found a significantly higher 7-day mortality in patients aged 80 and above. These patients were more frequently diagnosed within the ICD-10 Chapter IX, Circulatory Diseases. The patients were transported to a hospital with an MECU physician escort (44.1%) almost as often as they were transported by ordinary ambulance personnel (45.6%). This was by far the highest rate of physician-assisted transport in any age group. This indicates that convulsions in octogenarians in itself is considered an indicator of severe disease.

### Cardiac arrest

Another study has shown that 4.3% of patients with out-of-hospital cardiac arrest present with convulsions [45]. In our cohort of 3,388 patients presenting with convulsions, 45 (1.3%) experienced cardiac arrest either prehospitally or upon hospital arrival. These findings suggest that while convulsions may occasionally be associated with cardiac arrest, the overall incidence is low. Therefore, convulsions alone may not serve as a reliable prehospital indicator for cardiac arrest. Further research is needed to determine whether convulsions can be integrated into early screening protocols for cardiac arrest in the prehospital setting.

### Strengths and limitations

This study was conducted within a prehospital system that is based on EMTs and paramedics, supported by an on-scene physician [16]. This improves prehospital diagnostic accuracy, as a trained doctor assesses the patient before arrival at the ED. This strength, however, limits the generalizability of the study to countries with different prehospital systems.

Unlike other prehospital systems, the Danish system prioritizes patients with convulsions with the highest level of dispatch in each case. This leads to a very high sensitivity in including patients with convulsions in this study. However, in some cases, when the ambulance reaches the patient before the MECU, the issue may already be resolved due to possible simple convulsions, and no physician assistance is needed, resulting in the MECU being waived before arrival at the scene. Any convulsing patient treated solely by the personnel in the ambulances thus evaded inclusion into the study, seriously reducing the generalizability of our results to non-physician-staffed prehospital systems.

A significant strength of this study is its large sample size. With 3,388 records over 10 years, this register-based study is, to our knowledge, the largest study conducted on patients presenting with convulsions prehospitally. Another strength of this study is the thorough inclusion process. The manual review of 5,440 chart entries that merged into 3,388 individual patient courses ensures that a patient could be identified in multiple ways, underscoring that all eligible patients with high probability have been identified in this study. In this respect, we consider the exclusion of 66 contacts (1.9% of all potentially eligible patients) due to missing or obviously erroneous data to be a minor limitation of the study.

The predominant use of in-hospital assigned diagnoses in establishing the final diagnoses allows for much greater diagnostic accuracy, as the diagnostic tools available in-hospital and the longer observation times applied within the hospitals probably result in more accurate diagnoses than those assigned in the prehospital setting.

The high number of non-specific diagnoses assigned to patients, however, weakens this study by reducing data precision and potentially introducing misclassification bias.

## Conclusion

In this retrospective cohort study, we correlated patient age with the underlying cause in convulsing patients. The underlying cause for convulsions in prehospital patients varies greatly depending on patient age. This results in significantly different patient outcomes depending on patient characteristics. Insight into the shift in underlying causes and their severity related to patient age may help the prehospital physician determine the correct course of action for the patient, benefiting both the provider and the patient.

## Data Availability

Anonymized data are available from the corresponding author on reasonable request.
